# On the Law of Certainty

**Published:** 1863-07

**Authors:** W. G. Davies


### Art. II.—ON THE LAW OF CERTAINTY.

By the Rev. W. G. Davies.

That disquisitions on psychological topics should, to a great many persons, be devoid of interest, as having no moral or practical result, is one of the strongest evidences of the need of such inquiries, and betokens the undue sway which physical science, the inferior grade, the democracy of knowledge, exercises among us. To imagine that psychological inquiries are unprofitable speculations—a mere juggle of words, and good only to sharpen the intellect—is to exhibit the grievous ignorance which is so prevalent on matters intimately connected with the progress of the human mind. It has been remarked, but with just sufficient truth for a jest, that on metaphysical subjects there are more writers than readers. That there is, indeed, no adequate psychological public is manifest by the slow sale of works on psychology, and by the few and generally insufficient reviews which appear thereon. With few exceptions, professional critics do not seem up to the mark, and so, perhaps to conceal their ignorance, altogether slight an infinitely important study, or notice it only to condemn. As Wordsworth has done for himself, psychological writers must, in fact, create a public for themselves. At present it cannot be said that there is one. Inquirers are scattered and few. Yet how desirable is it that there should be such a public? We repose in the plenitude of our fancied knowledge, while the signs of the times, so forcibly manifested, indicate the downfall of long-revered dogmas, and in their room the prevalence of learned or Socratic ignorance. Great will prove the benefactor who shall lead men out of this ignorance into the light of new and elevating knowledge. "The end of philosophy," says Sir William Hamilton, "is truth, and consciousness is the instrument and criterion of its acquisition." Now the disregard in which philosophy is too generally held is not so much because there is not a considerable and increasing interest felt in abstract truth, as in consequence of a prevailing idea that psychology and systems of logic rather serve to mystify and sophisticate, than to enlighten, improve, and aid the mind. That there is great difference of opinion among mental philosophers is true. This, however, cannot be avoided, so long as our acquaintance with the human mind remains one-sided and incomplete. The inference, however, which is sometimes drawn from this is simply ridiculous. It is like judging Christianity by the life of some of its unworthy professors, or like pronouncing the science of medicine impossible, because there are diverse systems in vogue. Such inferences indicate narrowness, littleness, and indolence of mind. One thing is certainly manifest: there never was a greater amount of convergence among psychologists than there is at the present moment.

To that system of philosophy which presumes that the universal convictions of man are veracious, or accordant with the intention of the All-wise Creator, the system which from successive inquirers has received ever fresh advancement, especially is due the attention of those who, with an ardent love of truth, yet look upon metaphysics with suspicion and distrust. He who loves truth for its own sake, although he may have very false notions of the nature of true philosophy, deserves our respect. The man, however, who pooh-poohs the sacredness of the true, the fair, and the good, and with whom these are simply names for popularity and profit, is manifestly not to be won over to the cause. When, indeed, the crowd applauds, then and not before will he deem it impolitic to sneer. In the present inquiry I purpose building on the above-mentioned basis; and in furtherance of this object shall endeavour briefly to describe Reid's system, with its subsequent development by Sir William Hamilton, and what it further owes to the labours of Mr. Spencer Herbert.

Reid appeared at a time when the idealism of Berkeley and the scepticism of Hume had brought discredit on the current philosophy of the day. Uncertainty being for a time on the ascendant, Reid came forward as the champion of certainty. Berkeley and Hume having by reasoning, as Reid thought, arrived at their incredible conclusions, he very naturally insisted that all reasoning must repose on data, and that some of these in the outset must be prior to the exercise of reason. There are therefore, he said, truths which, carrying their own evidence along with them, necessitate their own conclusion. Of this character, so Reid protested, were the truths with which idealism and scepticism came into collision—namely, that there is an external world existing independently of man, and that our impressions and ideas have a substratum which is called mind. The existence of these entities, Reid maintained, could neither be proved nor disproved by reasoning, for they are known by common sense, simple feeling, or belief, native to every mind of man, and therefore common. They are consequently prior to all reasoning and of superior authority to reason. The attempt, therefore, to overthrow these primordial and universal beliefs by argument, he regarded as a mark of a badly constituted mind, of metaphysic lunacy. The root of our knowledge, he maintained, is faith; and the strongest plea which can be urged in favour of the authority of this faith, is the universality, inexplicability, and irresistibility of its character.

One obstacle, however, to the reception of Reid's system was the ideal hypothesis, or as Sir William Hamilton has more fittingly styled it, the representationist theory of outward perception. In Reid's day it was commonly held that the external world was known through the medium of a *tertium quid* which represented it to us, and that we had no immediate knowledge of material reality in itself. Here was a hypothesis opposed to our fundamental beliefs, which led us to regard the known object as the external object itself, and not simply a representation of it. Reid maintained this deliverance of common sense against the ideal hypothesis, which he held had no grounds whatever to rest upon, and therefore must be false if opposed to a primary conviction; and his peculiar merit as a philosopher he considered to be the refutation of the ideal hypothesis. This hypothesis, then, once fairly cleared away, the original data of consciousness, he thought, would have their high authority wholly unopposed. That Reid's position, in so far as it vindicates the veracity of consciousness, forms the only legitimate and lasting basis of philosophy, is my firm conviction. Its defects are those which are unavoidably attendant upon an incomplete analysis. Reid, like Wordsworth in poetry, returned to nature; and with a laudable dread of hypothesis, hasty generalization, and undue simplification, stated only what he reckoned to be facts. If he had thus only cleared the way for a new foundation he did good service, and if he did no more it is no mean service; for we must ever bear in mind that it is not given to one, but to many inquirers, to construct truth's temple upon an enduring base.

The philosopher who has been the first in advancing Reid's system to any extent is Sir William Hamilton. In his works, indeed, it reaches the stage of a healthy and promising vitality. Agreeing with Reid in the main, he yet gives to that philosopher's system a clearness and a fulness which Reid, engaged as he was in refuting error and vindicating the integrity of our primary convictions, could not impart. In relation to Hume, however, Sir William shows that Reid, who did not detect the distinction which subsists as between practical and reflective doubt—the doubt of the man and the doubt of the philosopher—carried his objections to the "sceptical system" too far. The common sense philosopher asks why Hume, since he doubted the existence of matter on the one hand, and of mind, the substratum of impressions and ideas, on the other, did not, as in consistency Reid thought he ought to have done, also question the existence of impressions and ideas? "The author of the treatise on Human Nature," says Reid, "appears to me to be but half a sceptic. He hath not followed his principles so far as they lead him; but after having with unparalleled intrepidity and success combated vulgar prejudices, when he had but one blow to strike, his courage fails him, he fairly lays down his arms, and yields himself a captive to the most common of all vulgar prejudices—I mean the belief of the existence of his own impressions and ideas. I beg, therefore, to have the honour of making an addition to the sceptical system, without which I conceive it cannot hang together. I affirm that the belief of the existence of impressions and ideas is as little supported by reason as that of the existence of minds and bodies. No man ever did or could offer any reason for this belief."* Now, Sir William Hamilton defends Hume against these strictures, and herein departs from Reid's system. To doubt the existence of impressions and ideas was, he shows, not attempted by Hume, because it is impossible to do so. "In Reid's strictures on Hume," says Sir William, "he confounds two opposite things. He reproaches that philosopher with inconsequence in holding to the belief of the existence of his own impressions and ideas. Now, if by the existence of impressions and ideas, Reid meant their existence as mere phenomena of consciousness, his criticism is inept, for a belief of their existence as such phenomena would have been a suicidal act in the sceptic."† Carrying out the same principle, he elsewhere says:—"Here at the outset it is proper to take a distinction . . . the neglect of which has been productive of considerable error and confusion. It is the distinction between the data or deliverances of con-

---

* Hamilton's Reid, p. 129. † Reid's Works, p. 129, note.

sciousness considered simply *in themselves as apprehended facts or actual manifestations,* and those deliverances considered *as testimonies to the truth of facts beyond their own phenomenal reality.* Viewed under the former limitation, they are above all scepticism. For as doubt is itself only a manifestation of consciousness, it is impossible to doubt that what consciousness manifests it does manifest, without in thus doubting—doubting that we actually doubt—that is, without the doubt contradicting, and therefore annihilating itself. Hence it is that the facts of consciousness, as mere phenomena, are by the unanimous confession of all sceptics and idealists, ancient and modern, placed high above the reach of question. Viewed under the latter limitation (*i.e.* considered as testimonies to the truth of facts beyond their own phenomenal reality) the deliverances of consciousness do not thus peremptorily repel the possibility of scepticism."* Here we see that Sir William advocates two systems; the one a rationalistic system, and the other a system of common sense. The ultimate authority for the reality of the phenomena of consciousness is reasoning—namely, that it is impossible to doubt the fact of the testimony of consciousness, or of the existence of the witness, without committing a suicidal act, a *subversio principii.* This is the *reason* given why the phenomena of consciousness are to be considered as being placed high above the reach of question. The ultimate authority, however, for the truth of what the witness declares, is said to be common sense, belief, or faith. The original data of consciousness, since they are primordial, are inexplicable, cannot be resolved into anything beyond. They must consequently be accepted on their own authority as simple feelings, beliefs, or trusts. Being at the root of all knowledge, they must, in the first instance, be received as valid. We have no choice afforded us but to begin with the presumption that our original beliefs are trustworthy; for the way in which consciousness starts into being is in the character of such beliefs. Without them, therefore, it is impossible to be conscious, and whether credited or discredited they will still assert their authority. Let what may, then, be thought of them in the second place, *i.e.*, after reflective observation, they must, in the first place, be attended with an implicit reliance in their integrity. There is no other alternative. Now, the obvious impression derived from this fact is, that these deliverances of consciousness cannot be mendacious. Is it, indeed, credible that the God of Truth, unless the humiliating fact were proved, would keep us all our days in bondage to a lie? In Sir William Hamilton's forcible

---

* Reid's Works, pp. 743, 744.

words—"To suppose the falsehood of our primary convictions is to suppose that we are created capable of intelligence in order to be made the victims of delusion, that God is a deceiver, and the root of our nature a lie."* "Nature is not gratuitously to be assumed to work, not only in vain, but in counteraction of herself; our faculty of knowledge is not, without a ground, to be supposed an instrument of illusion; man, unless the melancholy fact be proved, is not to be held organized for the attainment and actuated by the love of truth, only to become the dupe and victim of a perfidious creator."† This argument, which would be inadmissible in a rationalistic system, has its full force in the system of common sense, in which faith is made the root of reasoning.

Sir William, in the next place, shows how it is conceivable that we might bring discredit upon our spontaneous convictions. They are many. They can be compared together. If on comparison they shall be found mutually contradictory, either immediately in themselves or mediately in their necessary consequences, their mendaciousness will be proved. But Sir William is careful to declare that "no attempt to show that the data of consciousness are . . . mutually contradictory has yet succeeded, and the presumption in favour of the truth of consciousness and the possibility of philosophy has therefore never been redargued. In other words, an original, universal, dogmatic subversion of knowledge has hitherto been found impossible." This, then, is the position which Sir William Hamilton holds in respect to abstract truth. If considered in any other than a provisional light, it is questionable whether this constitutes a legitimate position. If one half of philosophy be capable of development into a rationalistic stage, this seems to augur that the other half will be found capable of a similar development. In the meanwhile, however, no more steadfast philosophic stand can be made than on common sense, or on the integrity of consciousness.

Mr. Spencer Herbert, seeking a datum for all certainty, after stating his opinion that Reid failed to disarm scepticism, and did little more than utter a prolonged protest against it, acknowledges that Sir William Hamilton has placed the common sense philosophy on a more secure footing, but thinks that he has failed to render it criticism-proof. He then objects to the test which Sir William proposes, namely, the detection of any mutual contradiction among the primary data of consciousness. Of this he says, "The test simply fails to help us, the only harm being that the offer of a valueless guarantee lays open to

---

* Reid's Works, p. 743. † Ibid., p. 745.

cavil that which it is put forward to insure."* In so far as Sir William declares that the mendacity of consciousness would be *proved* were its data found mutually contradictory, he certainly lays himself open to criticism. To this Mr. Herbert justly objects, that to suppose a proving power capable of proving the mendacity of consciousness in general, is to make that power prove its own want of veracity. If Sir William had said that the trustworthiness of consciousness would be rendered doubtful were its data found mutually contradictory, he would have been more correct, for we can imagine that the sceptic, in that case, would be bewildered, deeming it equally without show of reason why he should in the last resort, that is, reflectively but not practically, credit anything at all, even to the fact of his not crediting anything. He could not believe, he could not disbelieve, since that involves believing. He would be tossed about like a log on a sea of uncertainty. Now Hume might have carried his scepticism to this length, but expressly avoids doing so, because he did not see the utility of it, and still have answered to Reid's criticism: True, in one sense, I cannot doubt the existence of impressions and ideas, nor can I, any more than yourself, avoid feeling that there is an external world. As a man, I have the same convictions as all other men have, but as a philosopher, as a reflective inquirer, what I fail to find is any reason why the original data of consciousness should be veracious. For why may they not be all that they are, even although there be no such objects as they seem to reveal to us?" As it was, Hume did not carry his scepticism so far. He, however, because he could detect no evidence of power in causation, and because the ideal hypothesis was in conflict with the fundamental convictions of mankind, went so far as to give the preference to neither horn of the dilemma, as Berkeley did to the idealistic, and Reid to the realistic horn; and to expose, with something like a sneer, "the whimsical condition of mankind, who must act, reason, and believe, though they are not able, by their most diligent inquiry, to satisfy themselves concerning the foundation of these operations, or to remove the objections which may be raised against them."†

In criticising Sir William Hamilton's test, however, we must bear in mind that his motive in bringing it forward is to show that the only possible way to indicate the mendacity of consciousness has hitherto proved unsuccessful, and that the integrity of our intelligence has ever been unimpeachable. Now

---

* *Principles of Psychology.* Introduct., p. 9.

† Hume's *Essays.* An Inquiry concerning Human Understanding. Part I. Sect. xii.

I am of opinion that Sir William's test, so far as it goes, is effectual. It is purely negative, and as such is virtually complete. The primary data of consciousness must, in the first place, be relied on as trustworthy. There is only one way in which it is possible to bring discredit upon them. But no attempt to bring discredit upon them in this one way has ever met with the slightest success. Since, therefore, there is no room whatever for doubting the integrity of the primary data of consciousness, and since to doubt their integrity without reason is absurd and a madman's act, the belief that the God of Truth is not indeed the creator of a lying intelligence acquires overwhelming force. This, in opposition to Mr. Herbert, I must regard as at least one effectual answer to scepticism.

It becomes necessary here to remember that the above is not the only rejoinder to scepticism which Sir William makes. If, in round terms, we divide the realm of knowledge into the subjective and the objective, that which knows and that which is known, we must bear in mind that doubt as to the former is shown by Sir William to be impossible. Mr. Herbert takes no notice of this rejoinder; why I cannot conjecture, except it be that he thought his views when propounded would suggest so manifest a refutation of it that a formal criticism would be needless. The view I take of this rejoinder is, that it is a decisive reply to practical doubt, but to that reflective doubt which demands evidence of the trustworthiness of our intelligence in general, and therefore of Sir William's reasoning as a test, it yields no satisfaction. We must, however, postpone this point till we get further on. And now for Mr. Spencer Herbert's system.

Mr. Herbert sets out in search of a datum. This he states must not be any truth in which belief is reposed, but a canon of belief itself. Compare all truths together, and they agree in this—that they form objects of belief. The first principles of Reid were so many objects of this character. This, however, is not what we want, but rather a description of that state of mind which these data call forth, in short, a canon of belief.

Belief Mr. Herbert considers the fundamental fact, that which underlies all others. It is only as we believe that we exist, that we exist as *ad nos*. It is only as we believe that we are conscious that we are conscious. It is only as we believe that we have ideas, that we are possessed of them. In Mr. Herbert's own words:—"Hence, belief is the fact which to our intellects is antecedent to, and inclusive of, all other facts. It is the form in which every fact must present itself to us, and therefore underlies every fact. It alone of all things cannot be denied without direct self-contradiction. The propositions—

there is no consciousness, there are no ideas, there is no personal identity, may be absurd ; but they are not immediately self-destructive. To say, however, there is no belief, is to utter a belief which denies itself."* Having made out that belief is the ultimate fact which cannot be transcended, Mr. Herbert, in the next place, proceeds to a classification of beliefs. He divides them into variable and invariable. By a variable belief he means one of which the negation is either realizable in fact or conceivable as a supposition. By an invariable belief he means one the negation of which it is not possible for us to conceive, one which we cannot change if we would. "We see first," he remarks, when recapitulating his views, "that belief is fundamental, and that the invariable existence of a belief is our highest warrant for it; second, that we can ascertain the invariable existence of a belief only as we ascertain the invariable existence of anything else, by observing whether under any circumstances it is absent from the place in which it occurs; third, that the effort to conceive the negation of a belief is the looking in the place in which it occurs (viz. after its antecedents), and observing whether there are any occasions on which it is absent, or can be made absent; and fourth, that when we fail to find such occasions—when we conceive that the negation of the belief is inconceivable, we have all possible warrant for asserting the invariability of its existence; and in asserting this we express alike our logical justification of it and the inexorable necessity which we are under of holding it. Mean what we may by the word truth, we have no other choice but to hold that *a belief which is proved by the inconceivableness of its negation to invariably exist is true.* We have seen that this is the assumption on which every conclusion whatever ultimately rests. We have no other guarantee for the reality of consciousness, of sensation, of personal existence, we have no other guarantee for any axiom, we have no other guarantee for any step in a demonstration. Hence, as being taken for granted in every act of the understanding, it must be regarded as the Universal Postulate."†

It is scarcely necessary to state that all our beliefs are not fundamental. The ultimate warrant, however, for the validity of any belief is the invariableness of its existence as evidenced by the inconceivableness of its negation. Thus, in a series of beliefs, one depending upon another, the highest guarantee for a belief is also the invariableness of its existence. Or, to take another view of the case, if we assign as the proof of a belief

---

* *Principles of Psychology.* Introduct., p. 15.
† Introduct., pp. 30, 31.

an ulterior belief on which it depends, it is evident that we cannot do this without end, but must eventually come to a fundamental belief which, carrying its own evidence, necessitates its acceptance on its own authority, and that the only thing we can say of it is, that it is invariable.

Another principle to which prominence is given by Mr. Herbert is, that the more fundamental a belief is, the more certain it is, because the liability to error is not so great in the case of a single belief as in the case of a long chain of beliefs. The liability to mistake is far greater when a lengthy column of figures is added up than when we simply add two units together. The application made of this principle by Mr. Herbert is important. An original datum of consciousness, such as the belief in the existence of the external world, being simple and invariable, is superior in certainty to the more complex combination of beliefs with which idealism and scepticism attempt to overthrow it, because the liability to error of a complex combination is so much greater than the simple primordial belief to which it is opposed. No objection framed by idealist or sceptic can therefore be so certain as a primordial belief—an original datum of consciousness.

While admitting that Mr. Herbert has cast additional light upon this important subject, I must confess that I do not think he has exhausted all that can be said upon it. In some respects, too, it seems that he is incorrect. For instance, I do not see that belief is the best word which could be selected in order to designate the fundamental datum. Belief, both in the popular and the theologic sense, is distinguished from knowledge. I believe you are right, and I know you are right, are expressions to which no one in ordinary conversation attaches equal force. The first implies an opening for doubt, the second does not, and in religion the completeness of faith consists in the moral victory gained over this doubt. I am aware that in scientific language terms have a technical meaning, and that they must not in that case be understood in their wider sense. It is clear, however, that the word belief, when it was applied to an original datum of consciousness, was meant to convey the fact that it was to be accepted as true on its own authority as a simple feeling or trust. "St. Austin," according to Sir William Hamilton, "accurately says—we know what rests upon reason, we believe what rests upon authority. But reason itself," continues Sir William, "must rest at last upon authority, for the original data of reason do not rest on reason, but are necessarily accepted by reason on the authority of what is beyond itself. These are, therefore, in rigid propriety, beliefs or trusts. Thus it is that in the last resort, we must perforce philosophically admit that belief is the primary condition of reason, and not reason the ultimate ground of belief. We are compelled to surrender the proud *Intellige ut credas* of Abelard, to content ourselves with the humble *Crede ut intelligas* of Anselm."* Now, in a system of common sense, and that more especially as Reid and Stewart left it, there is sufficient justification for this phraseology. They, as it were, preached faith in the fundamental deliverances of consciousness, insisting that no evidence should be sought in respect of the trustworthiness of these primary revelations, for the very plain reason that they are primary and therefore inexplicable. Belief was just the word that their system demanded —no other was so appropriate. When, however, we turn to Hamilton, and discover that as relates to the subjective he has, as he professes, proclaimed the impossibility of scepticism, we must also infer that he has, in so far, translated the subjective out of the domain of faith into that of knowledge. For, to show that a deliverance of consciousness is proof against scepticism is, if the word has any meaning distinct from that of belief, to arrive at knowledge of the purest order. Again, as regards the objective, in so far as he places the original data of consciousness on an unassailable elevation, so far does he render them objects of knowledge rather than of belief. It is, however, only fair to state that Sir William considers the argument from the common-sense point of view, as it enables us to vindicate the truth of the phenomena of consciousness, viewed as attestations of more than their own existence, as not placed beyond the possibility of doubt. In this view of them, to call the original data of consciousness as vouchers for more than their own existence, beliefs, or trusts, is quite consistent. We must, however, insist that he has intentionally done his best to eliminate that element of doubt which gives to these terms their peculiar appropriateness.

I would remark in this place that the philosophers of the Scottish school, by a primary belief, mean not simply what is expressible in a particular proposition, as matter of fact, but also what is expressible in a universal proposition, as, for instance, axioms and definitions. Now, they have in this theory strong grounds for insisting that belief is the primary condition of reason. For, if primary universal propositions be got intuitively, faith must prove to be their origin. If, however, primary universal truths turn out to be inferences from particular premisses, belief ceases to occupy the position demanded for it by the Scottish philosophers. This, it is hoped, will become more evident the further we proceed with this inquiry.

* Hamilton's Edition of Reid's Works.

But how is it with Mr. Spencer Herbert, who rejects Sir William Hamilton's test as not criticism-proof, and substitutes one of his own, which he deems unassailable? Is his position impregnable? Then why does he use the word belief to designate the fundamental datum which by Reid, Stewart, and Hamilton is employed, because our common-sense deliverances, in regard to what they testify, are confessed to be open, as idealism and scepticism prove, to question? If, however, Mr. Herbert have utterly annihilated scepticism, I cannot see why, after achieving this success, he should call the fundamental fact belief. By all means make belief the stepping-stone to knowledge, but after we have acquired knowledge, cease to call it belief.

I have also considerable difficulty in reference to another tenet of Mr. Herbert's. He says that belief is more fundamental than consciousness, that belief is the primordial fact. "It alone," he asserts, "of all things cannot be denied without direct self-contradiction. The propositions—there is no consciousness, there are no ideas, there is no personal identity—may be absurd, but they are not immediately self-destructive." Strong assertions these, but are they true? Let us take them in the order in which they stand. There is no consciousness. This, when expanded into the concrete form, which is a sure way of testing its validity, must assume the following shape—I am conscious that there is no consciousness. This is a clear contradiction. Again—there are no ideas. This, when fully stated, must mean, I have an idea that there are no ideas. And since ideas are simply states of consciousness, we have as before the *reductio ad absurdum*—I am conscious that there is no consciousness. Again—there is no personal identity. This, when expanded, must mean—I am conscious that I exist not, a proposition in which the *ego* is posited in the subject to be denied in the predicate. Mr. Herbert's main position is undoubtedly wrong—there can be no belief without consciousness. Consciousness connotes belief as one of its essential elements in the same way as the word water connotes oxygen. But as oxygen is not more fundamental than hydrogen in the constitution of water, so belief is not more radical than the other essential attributes of consciousness. Belief is one *sine quâ non* of consciousness, the cognition of an object is another, consequently, to fix upon belief as more fundamental than an attribute equally as essential, and therefore equally as radical, is wholly arbitrary. For why not say of a triangle, that to be equiangular is more fundamental than to be equilateral? To say of a whole, especially a primordial whole like consciousness, that one essential attribute is more fundamental than another essential attribute, is to imply that consciousness can exist *minus* a condition of its existence. None of the elements of consciousness have an existence prior to that of the complex whole which consciousness is discovered to be. Instead, therefore, of saying that belief is the fact which underlies every other, the truth is, that as *ad nos*, there is no more fundamental entity than consciousness in its totality, and the conditions of its existence are of two kinds, intrinsic and extrinsic. Among the first we may mention cognition and conviction; among the second an object. But here it is necessary to remark that it is impossible to fix upon any one term which shall express some one element of consciousness and no more. For instance, cognition implies conviction, and conviction implies cognition. The same is true of belief. All that we can expect from such terms is, that one of them should give prominence to this element, rather than that. That Mr. Herbert has carried abstraction too far becomes evident also from this, that after distinguishing between consciousness and belief, and asserting the priority of the latter, he is afterwards obliged to recall the abstracted entity, and to use such expressions as these—"Though in common language we speak of a belief as something separate from the conception to which it relates, yet on analysis we find that we simply express by it a certain property of such conception, *its persistence*." "The belief is not something more than the state of consciousness, but merely expresses its persistence."* This is like publicly thrusting a man out of an office to be under the necessity of privately recalling him. What Mr. Herbert ought to have said is, that belief more particularly designates that state of consciousness in which consciousness best admits of being regarded as the fundamental datum, the universal postulate.

It is rather amusing to see how some writers, seemingly in total ignorance of the teachings of the most advanced psychologists of the day, degrade the word consciousness into meaning simply the cognition of internal phenomena, or, as Mr. Bain* does, into signifying the merest germs of cognition, as if it were not plain that by consciousness is meant knowing in general. Consciousness is the *summum genus*, which includes every, even the most complex, intellectual act. Mr. Bain, in attempting to deny that consciousness is the standard of truth, traces our knowledge to its very germs, and then denies that these, except in the most limited sense, are the standard of truth, as if any one were so absurd as to maintain anything else. When it is said that consciousness is the standard

* Introduct., p. 30.

* *The Emotions and the Will.* p. 557. sec. 10.

of truth, it is meant that the several operations of thought which the words denotes are each of them the sole criterion of its related truth. If, then, our intelligence is not the standard of certainty, I should like to know what is.

Now, allowing that belief, assurance, conviction, aptly designate consciousness as a fundamental fact, is it not undeniable that conviction is subordinate to evidence? This Mr. Herbert implies when he makes use of the words "antecedents of belief." If belief has antecedents, it must be consequent on the same, and they, not it, must be the fundamental fact. Nay, without belief, Mr. Herbert will reply, these antecedents could have no existence, for it is only in so far as we believe in their existence that they can possibly exist relatively to us. Then why are they called antecedents? I do not ask this question to convict Mr. Herbert of a mistake. I also hold that they are antecedents. But see the consequences of this admission. Belief is antecedent to its antecedents, and its antecedents are antecedent to belief. But is not this a contradiction? Properly understood, it is not. Mr. Herbert is right in maintaining that belief is prior to its antecedents, but it is also perfectly correct to hold that its antecedents are, as the term signifies, prior to consciousness. Here is a mystery, how is it to be solved? Let us call the antecedents of consciousness evidence. The problem which we have to solve will then assume this shape—consciousness is prior to evidence, but evidence is also prior to consciousness. Now, it is manifest that these two statements cannot, in the same sense, both be true, because they are mutually subversive. Let it be understood, therefore, that there are two orders—the order of knowledge and the order of existence. In the first order, consciousness stands between us and all being—the being of the world, the being of God, our own being, yea, and the being of consciousness itself, which only exists in so far as it knows itself to exist. We have but one access to being, and that is through the medium of consciousness, knowing, or intelligence. Consciousness, therefore, relatively to us, is antecedent to existence, is, as Mr. Herbert rightly teaches, the fundamental fact. I had good reasons, therefore, for stating above—if our intelligence is not ths standard of certainty, I should like to know what is. But now comes the counter order. Consciousness avers the existence of objects. It also declares that, in the order of existence, the object is prior to consciousness, and that there can be no consciousness without an object. If consciousness, therefore, is trustworthy—for all depends on this, and for the present we must postulate that it is so—it is quite true that evidence, in one sense, is antecedent to conviction. If, therefore, in the order of knowledge, the order of idealism, being *minus* consciousness, is impossible, so also in the order of existence, the order of realism, consciousness *minus* being, is equally as much an impossibility, an impossibility in three senses; first, in so far as consciousness is itself a being or entity; secondly, in so far as it it cannot exist without the man, who is also a being or entity; and thirdly, in so far as it cannot exist without objects to take note of—that is, entities to call it into action. In the *cogito ergo sum* of Descartes we have a compendious mode of expressing the order of knowledge. The converse of this position —namely, *sum ergo cogito*—not less compendiously expresses the order of existence.

Now, while it cannot be denied that Mr. Herbert has advanced the law of certainty in the order of knowledge, it still remains open for some one to develop that law into fuller proportions in the order of existence. It is quite true that the state of consciousness variously termed conviction, assurance, certainty, positiveness, and so forth, is primordial in the order of knowledge, and we are willing to adopt most that Mr. Herbert teaches on this head. If, however, assurance is subordinate to evidence, it must be an advantage to know the various kinds of evidence which determine our convictions. The end of philosophy is abstract as well as concrete truth, and it more effectually fulfils its office when, by describing the essence of certainty, it enables us to identify truth when we meet it, than by simply testing the truths of which we are already possessed in a spontaneous or empirical fashion. It would, however, lead to far too great a length in this place to give, were it only a general, description of the truth-acquiring powers of the mind, and the appropriate evidence which each of them requires in order to bring it from a state of uncertainty to one of certainty. I shall, therefore, simply content myself with discussing the evidence of that class of truths which constitute science—namely, necessary and universal truths.

Mr. Herbert, in criticising Mr. J. S. Mill's views in relation to Dr. Whewell's test of necessary truth, has the following passage, highly suggestive, indeed, of the doctrine which I am about to propound. "If there be, as Mr. J. S. Mill holds, certain absolute uniformities in nature, if these absolute uniformities produce, as they must, absolute uniformities in our experience, and if, as he shows, these absolute uniformities in our experience disable us from conceiving the negation of them, then, answering to each absolute uniformity in nature which we can cognise, there must exist in us a belief of which the negation is inconceivable, and which is absolutely true. . . . . In nearly all cases, this test of inconceivableness must be valid now, and where it is not, it still expresses the net result of our experience up to the present time, which is the most that any test can do." Mr. Herbert declares, then, that what is contrary to absolute uniformity of experience is inconceivable, and that this is the only test of the invariableness of a belief. Is a belief invariable? We know that it is so by the inconceivableness of its negation. And why is its negation inconceivable? Because it is opposed to our unbroken experience. But is this test absolutely perfect? Is it possible that a belief, proved by it to be invariable, should ever turn out to be otherwise than invariable? Mr. Herbert admits that his test does not preclude this possibility. Indeed, how can uniform experience prove the impossibility of its non-uniformity? No induction from such experience is competent to establish a necessary and universal truth, and it is only the negation of such a truth which is *per se* inconceivable. In the controversy between Mr. Mill and Dr. Whewell, in regard to inconceivableness as the test of necessary truth, it is contended by the former, owing to an *error personæ*, that certain beliefs were once held to be true, because their negation in some sense was inconceivable, which beliefs are now exploded, and therefore that inconceivableness is no infallible criterion. But the inconceivableness which Dr. Whewell has in his mind is that which we experience when we try to think the contrary of a necessary truth, as, for instance, that $5+5$ does not make 10. The inconceivableness which Mr. Mill selects is that which certain men feel when they attempt to undo firm but ill-founded associations. Mr. Herbert weakens Mr. Mill's objection to Dr. Whewell's view, but does not detect its complete futility. He says: "Conceding the entire truth of Mr. Mill's position, that during any phase of human progress the ability or inability to form a specific conception wholly depends on the experiences men have had, they may, by-and-bye, be enabled to conceive things before inconceivable to them, it might still be argued that as, at any time, the best warrant men can have for a belief is the perfect agreement of all pre-existing experience in support of it, it follows that, at any time, the inconceivableness of its negation is the deepest test any belief admits of. *Though occasionally it may prove an imperfect test,* yet, as our most certain beliefs *are capable of no better,* to doubt one belief, because we have no higher guarantee for it, is really to doubt all beliefs."* By inconceivableness, then, Mr. Herbert means the same thing as Mr. Mill does, namely, that which is contrary to absolute uniformity of experience. While, however, Mr. Mill, in opposition to Dr. Whewell, makes little of the test, and exposes its weakness, Mr. Herbert makes the most of it, maintaining that it is the

* Introduct., p. 21.

best which we have. Is it the best? Twice two is four, and never by any possibility can be anything else than four. But how do you know that? Because the negative of the statement is inconceivable. But such a test, according to Mr. Mill, is of little weight, and even according to Mr. Herbert, who exalts it, as he thinks, to the utmost, is not infallible. If this, therefore, be the best test we have, it must be allowed that to say that twice two cannot by any possibility make anything else than four, is asserting more than we have a warrant for. We have a conviction which is stronger than our criterion; yet we feel positive that the conviction is not, and cannot be, too strong. Must not the test, therefore, be incompletely stated? Why does the chemist, when he has faithfully analyzed a specimen, conclude that he has ascertained, not only the elements of that particular specimen, but of the whole species of which it forms a part, and that irrespective of time? Or why does the reflective observer, when he has carefully analyzed some portion of his own consciousness, feel confident that he has discovered, in so far as his analysis is correct, a law of all human intelligence? It is according to the same law, in conformity with which we infer that twice two must always make four. If we merely had the evidence of invariable experience for the truth of this simple statement, Mr. Herbert's test would be the best which is obtainable. By invariable experience we must not, however, understand only what is positive, but also what is negative. We do not merely perceive that two and two always make four, but we also perceive that no other combination, or its equivalent, ever makes four. Still, however, the test, even when thus described, has an element of uncertainty in it; because human experience, whether positive or negative, or both, is limited. But in twice two is four we have a truth which is unlimited, which is universal. How are we to eliminate this weakness from the test? In some instances, it is pointed out by Mr. Mill that we have not the power, from the absence of any analogy, to imagine an exception to our uniform experience, we cannot, from want of material wherewith to do it, construct a notion which negatives the knowledge which we possess. We cannot, for example, imagine space which has no space beyond, time which had no time prior to it, or which will have no time posterior to it, or picture the character of elements simpler than those which baffle the attempts of analysis to resolve them into yet simpler ones. This explanation is ingenious, but not satisfactory. Inconceivableness arising from the want of material wherewith to construct a notion does not appear to constitute the test of necessary truth. In the great majority of truths of this class, we are not deterred from imagining exceptions to them by the obstacle which Mr. Mill mentions. For example, we are compelled to regard the law of causation as a necessary truth, yet Mr. Mill avows that it is quite conceivable to his mind that in some other sphere than this events may take place entirely at random. The inconceivability mentioned by Mr. Mill is analogous to the inability of a man blind from birth to form a notion of colours, or that of a child to experience connubial love, whereas the inconceivability of the negation of a necessary truth arises, not from impotence to imagine the negation, but from the perception of contradiction between the truth implicitly felt to be necessary, but explicitly capable of being proved such, and a merely imaginary negation of it. It seems, therefore, that the test of which we are in search is the following, namely, the Canon of Induction :—

Other circumstances being absent, if the existence of any set of circumstances is attended with the existence of another set, and the non-existence of the first set is attended with the non-existence of the second set, then the existence of the first set is necessary to the existence of the second set.

This canon seems to afford the criterion of necessary truth. According to it there is no alternative save for a conjunction among facts to be necessary or not necessary, that is, contingent. It has, however, always been held that a necessary truth is virtually universal. Now it appears that the universality of a necessary truth is inferred in harmony with the following law of contradiction, which I call the Law of Universalization :—

If the existence of this set of circumstances is proved necessary by induction to the existence of that set of circumstances, you cannot conceive an instance in which the first set is not necessary to the existence of the second set, without coming into collision with a demonstrated truth. Whereas, in conceiving instances in which the first is necessary to the existence of the second, you keep in harmony with such truth, and, consequently, the inference is rendered unavoidable that at any period, past, present, or to come, and in any number of instances, the first set of circumstances is necessary to the existence of the second set.

I have now, to the best of my ability, exposed the weakness of the test proposed by Mr. Herbert, and also exhibited the nature of that absolute inconceivableness which is experienced by those who would imagine the negation of a necessary truth. This is done by endeavouring to show what a necessary truth is, and that the inconceivableness of its negation arises from opposition to the evidence which determines its necessity.

The result at which we have now arrived is the following. If the origin of necessary and universal truths have here been rightly accounted for, there ceases to be any justification for opposing the *à priori* to the *à posteriori* method and the converse. In so far as we transcend experience, when from one or more verifications of the necessity of a truth we infer the universality of the same, when from one or some we proceed to all, the method pursued is *à priori*. Since, however, every truth must involve germs derivable solely from experience, and since every universal truth, so far as relates to these germs, must be uniformly verifying itself in experience, the method concerned must also be *à posteriori*. In consequence of this intimate union between these two methods, it follows that a host of truths, hitherto named contingent, are seen to be misnamed, and, as Dr. Whewell has already more than suspected, must be looked upon as being as much necessary truths as the truths of geometry and number. According to the Canon of Induction, the propositions, twice five is ten and all fire burns, are in the same category. Whatever evidence is cited for the necessity and the universality of the former, can also be cited for the necessity and universality of the latter. There is, however, one source of ambiguity attending this question which still demands attention.

Some have thought that there is no sufficient ground for making a distinction between necessary and contingent truth. What do we mean by these expressions? Any truth, if what it professes to be, is necessarily true. To say that it is contingently true, is to admit the possibility of its being untrue. A contingent truth therefore is necessarily true. It is, for instance, as necessarily true that a man's hands are in his pockets while they are there, as it is necessarily true that a whole is greater than its parts. What, therefore, is the precise meaning of the words, necessary truth and contingent truth? By a necessary truth I understand a necessary conjunction between one fact and another, and by a contingent truth a conjunction which is not necessary, that is, contingent. It would consequently be more correct to call a necessary or a contingent truth, a necessary or a contingent conjunction, connexion, or concatenation. The necessity or the contingency does not express the nature of the cognition, but the nature of that which is cognised. To use Mr. Herbert's phraseology, we have an invariable belief, both in a variable and in an invariable connexion of facts. When, according to the Canon of Induction, a conjunction is proved to be not necessary it is contingent. Consequently, necessity and contingency are related terms, for not necessary means contingent, and not contingent means necessary. The whole universe of conjunctions among facts is exhausted by these two terms, for every such conjunction is either necessary or contingent. This is a cardinal law of nature.

Sir William Hamilton contends that the only logical induction is that which infers the whole from the enumerated units which compose it,* and that reasoning from particulars to generals is extra-logical. If, however, it be extra-logical, by far the most important kind of reasoning does not come within the province of logic, a fact more damaging to the reputation of that science than the propounder of the above doctrine can have fully foreseen. But why is the Baconian or material induction said to be extra-logical? Because it has not been rendered formally valid, that is, such that its conclusiveness should appear from the formula which gives explicit expression to it. Now, if it be possible to render the Baconian induction formally valid, and this is done when it is expressed in harmony with the canon given above, it then claims a foremost, if not *the* foremost, place in the science of reasoning. Indeed, so strong is the presumption in favour of its capacity for rigid conclusiveness, that to reserve no place for it in pure logic required no small amount of devotion to a pet theory on the part of the objector, and should not fail to elicit the disapproval of the scientific world. In ultimate analysis, what are the primary data of any science? In the order of knowledge, they are invariable convictions. But what, then, is the criterion of their invariability? Why, inconceivableness of their negation. But whence does this inconceivableness proceed? From being opposed to the evidence which establishes the necessity of a conjunction between one fact and another, namely, induction according to the canon. We owe, therefore, the primary data of any science to Baconian induction. Consequently, to call this extra-logical only serves to bring the so-called pure logic into well-deserved disrepute. *The* pure logic, the logic of the future, will, we may be assured, give the leading place to material induction.

The very laws of thought evince, in a very marked degree, the necessity of maintaining the validity of the Baconian induction. Does not logical psychology, so ably elucidated by Dr. Mansel, in the *Prolegomena Logica*, display the necessary laws of thought, namely, those laws to which the intelligence of the whole human race is subject? And does it not challenge for these a precision and a necessity which it denies to the laws of the physical universe? But now, as to the basis on which logical psychology must be constructed. Mental science must arise from an analysis, in the first place, of one's own intelligence, and in the second, from observing the intelligence of other men, as it manifests itself by means of its appropriate signs. For it is only by reading these signs in the light of one's own consciousness that they convey the slightest meaning. Suppose the analysis to be confined to what we know at first hand, one's own consciousness. How are we, provided there be

* X Y Y are some B. X Y Z are all A. ∴ All A is some B.

no more fertile induction than the so-called formal, to be certain that there are necessary laws therein, or that what are necessary laws at present will be such for all time? And how are we to be certain that the same are the necessary laws of all human thought? It may be urged in reply that there are primary convictions, or self-evident truths, data accepted on their own authority as simple feelings or trusts. Or it may be said, in the words of Dr. Mansel, that "whatever truths we are compelled to admit as everywhere and at all times necessary, must have their origin, not without in the laws of the sensible world, but within in the constitution of the mind itself. Sundry attempts have indeed been made to derive them from sensible experience and constant association of ideas, but this explanation is refuted by a criterion decisive of the fate of all hypotheses, it does not account for the phenomena. It does not account for the fact that other associations, as frequent and as uniform, are incapable of producing a higher conviction than that of a relative and physical necessity only."* Now, what is Dr. Mansel's evidence for stating that truths which we are compelled to admit as everywhere and at all times necessary must have their origin, not without in the laws of the sensible world, but within in the constitution of the mind itself? Evidently the failure up to this time, to render the Baconian induction formally valid. Owing to this defect, another origin has been assigned to these truths. But what can be meant by saying that a necessary truth must have its origin, not without in the laws of the sensible world, but within in the constitution of the mind itself? All truth in ultimate analysis involves the assent of consciousness, and consequently must have its origin within in the constitution of the mind itself. For the acquisition of necessary and universal truth, however, uniform experience of the external world, although an *à posteriori* condition, is not sufficient. So far I agree with Dr. Mansel, but wholly differ from him as to the character of true induction, and its competency to supply us with necessary and universal truths. In furtherance of this view, let us examine one of the Kantian strongholds. "All analytical judgments," says Dr. Mansel, "are formed by the mind *à priori*, whether the notion analysed be empirical or not."† Now let the instance of an analytical judgment be, a whole is greater than its part. This is a necessary truth, and its necessity is said to arise, according to the law of identity, from the fact that there is nothing in the predicate but what was implied in the conception of the subject. But why, in such a case, must the subject imply or connote the predicate and that universally? What evidence is there on the point? Is it a primary convic-

---

* *Prolegomena Logica*, p. 99. † Ibid., p. 101.

tion that it does so, or rather are we constrained by the absence of any other alternative to think so? The conclusion to which I come is, that whenever there is a necessary conjunction between a subject and a predicate, it is because such a conjunction is established, either implicitly or explicitly by induction, between the facts expressed by them. And the grand result is, that we cannot establish necessary and universal conjunctions even among thoughts, on any other condition than that on which we must establish them among things through the medium of thoughts, our only access to things.

If, again, in the second place, we study human nature, at second hand, on a wide scale of observation, still our observation must be limited; and according to the Hamiltonian, or indeed any system of logic, incompetent to warrant a universal conclusion. Whence, then, come the superior certainty, necessity, and universality claimed for the laws of thought? The Hamiltonian logic demands for these laws a necessity and a universality for which it provides no formal guarantee, and simply refers us to the law of identity, the law of contradiction, and the law of excluded middle, as if these were possessed of final authority; but are they final? They are necessary and universal truths, and all truths of this class are derived, as it appears, from induction in accordance with the canon. The superior exactness, therefore, claimed for these laws of thought is an unfounded assumption, and I fail to see why the conclusions of material induction should not be accorded as great an authority as the facts of the thought-world itself. The striking defect in the Hamiltonian logic, here indicated, forcibly shows how inadequately it describes the operations of intelligence as they really are. And if at the very fountain-head of science the need of the Baconian induction thus prominently manifests itself, it would seem strange indeed were its *formal* validity to lie much longer in the dark.

Let us now take the parting view of this important subject. Let us suppose that the test given above is complete. If it be complete, it must be so because it leaves no opening for doubt. This, however, is simply true in relation to practical doubt. There is such a thing as reflective doubt, to which it still remains open. For instance, a theorem in geometry is satisfactorily demonstrated; can we then deny its truth? In a direct, as distinguished from a reflective sense, we cannot; but there still remains an opening for the question, Are the deliverances which constitute demonstration absolutely veracious? This may be an unreasonable question, but there is no contradiction involved in putting it. It is the sceptic's last objection; and although it borders on the frivolous, the answer to it sheds a flood of light on the subject before us. Besides, if the question be legitimate, and the answer to it can be given, it is better to give it, than to yield any further pretext to scepticism. In the first place, however, let us ask what portion of our intelligence requires this satisfaction. It is evidently not intuitive consciousness, for this possesses in the act of intuition all the evidence which it is competent to lay hold of. When a pen is handled, we possess the fullest evidence that intuition demands as to the existence of that pen, and any doubt cannot in the least weaken the intuition. Now that which asks whether this intuition be veracious, must clearly spring from some other portion of our intelligence than the intuition itself. This it seems is reason, and here again we are in opposition to Mr. Herbert. Reason, as the source of all reflective scepticism, seems to be the final arbiter in matters psychological. When all other faculties, and in a direct operation reason itself, are in a satisfied state, still reason in its reflex operation, if unsupplied with appropriate evidence, is sure to give vent to its craving in scepticism. And now as the annihilation of scepticism is, we may say, the final aim of philosophy, its ultimate office must be to supply reason with fitting evidence. Reason, consequently, must be the final criterion of certitude. Mr. Herbert broaches a doctrine opposed to this. He claims for our fundamental beliefs a superiority over all others, and considers the authority of reason weak in proportion to the remoteness of its data from those beliefs. His doctrine is stated thus :—"In proportion as the number of concepts which a proposition involves is great, and the mental transitions from concept to concept are numerous, the fallibility of the test will increase, and will do this *because the formation of the belief is separable into many steps, each of which involves the postulate.*"* "We hold it more certain that 2 and 2 make 4, than that 5 + 7 + 6 + 9 + 8 makes 35. We find that every fresh assumption of the postulate involves some risk of error; and, indeed, where the calculation is extremely intricate, and the assumptions therefore extremely numerous, our experience teaches us that the probability that there has been a wrong assumption is greater than that there has not. So, too, in argument. We lose faith in a long series of steps, however logical they may seem, unless we can test the inference by appeal to fact—that is, *unless we can get at the inference by a single use of the postulate.*"† That there is a certain amount of truth in this doctrine is clear enough, but it is overstated. Not only is it contrary to the method of nature, but also to fact, to state that the more complex a process of intelligence, the more fallible it is. Reasoning is more complex than intuition, but it is not on that account less trustworthy. According to the method of nature, the superior is that which presupposes

* Introduct., p. 32. † Ibid. p. 33.

the more general and simple. The Queen, as queen, presupposes both the existence and the loyalty of her subjects, whereas their existence is not dependent on that of any sovereign head. According to this method reason is superior to intuition, and asserts its superiority in scepticism—namely, in a demand for appropriate evidence. In philosophy, therefore, the *Intellige ut credas* of Abelard takes precedence of the *Crede ut intelligas* of Anselm. Greater liability to error on account of greater complexity, does not necessarily render reason less trustworthy. True, it is not so easy to add up a long column of figures as it is to add 5 to 5; but surely the result admits of as much correctness in the former instance as in the latter.

And now, what is the evidence which pacifies the final craving of reason? It is this. In the order of existence the final evidence of certainty is the fact that, according to the testimony of induction, veracity is presupposed both by proof and by disproof. The veracity both of intuition and of reason is involved in every attempt either to prove or to disprove the veracity of consciousness in general. For as you cannot arrive at truth by reasoning unless intuition supplies truth in the premisses, and unless reason draws a true conclusion from them, it is evident that if you attempt to establish the truthfulness of intelligence, you must do so by means of its truthfulness, which is a *petitio principii.* Or if you would disprove its truthfulness, you are forced also to do so by means of the very truthfulness which you attempt to overthrow, which is a *subversio principii.* Veracity therefore is, in the order of existence, the final fact of consciousness. In the order of knowledge the final fact is the invariableness of the conviction that the evidence here given fully substantiates the veracity of consciousness, that is, fully satisfies reason on the point, and affords it no further room for uncertainty. Hence it is that reason, and not common sense, is the final arbiter.

A great practical reflection arising from the result here attained is, that in regard to the facts of the mental world, he who boldly states them without hampering himself by any attempt at explanation, is more likely to be right than he who, in the early stages of research at least, analyses the workings of the mind, and admits nothing that has not a place in his analysis, incomplete and erroneous though it be. This shows us how much indebted we are to men like Reid, who with strong spontaneous convictions insist upon the existence of certain mental laws against those who, because they cannot explain them, attempt to do them away. How prone men are to make the narrow limit of their laborious research the limit of the knowable, the past too frequently testifies. Yet all honour to every earnest searcher in the realm of truth, whether one remain on the mountain-top or plod in the plain below. Close observation will yield to the plodder truths which the very largeness of view helps to conceal from the wider, which is to say, the less careful, observer. It is by the minute inquirers that truth receives advances towards the scientific or final stage. Wide observers meanwhile exercise a corrective and conservative function by checking the tendency into which the men of detail are apt to lapse of unduly magnifying their own sphere and slighting that of others. When Reid and Stewart, to use the words of the latter, held that "the belief which accompanies consciousness as to the present existence of its appropriate phenomena rests on no foundation more solid than our belief of the existence of external objects,"* they proclaimed the whole truth. If they had set themselves the task of verifying this truth by analysis, they could not have made a statement so bold, so comprehensive, and yet so correct. Of this we have abundant proof in the narrowness, one-sidedness, and exaggerated views of so many, especially the more early, rationalistic inquirers. Even Sir William Hamilton, great in speculation, greater still in learning, assails, in his otherwise praiseworthy efforts to reduce the laws of thought to their simplest form, the great datum of consciousness just quoted from Stewart. Mr. Herbert, when he claims for this datum a higher authority than can possibly be claimed for any reasoning that is brought against it, seems clearly to shoot ahead of Sir William. And now following the precedent set by the last-mentioned philosopher, when he declares that the phenomena of consciousness are far above the reach of question, because to doubt their existence is a suicidal act; I would likewise affirm, and for the same reason, that any deliverance of consciousness which is in accordance with the law of certainty is also far above the reach of question, both in relation to internal and to external facts. Thus the quotation from Stewart proves literally true, and common sense is found to be at one with the latest results of purely rationalistic philosophy. This proves the wisdom of making the *Crede ut intelligas* of Anselm the stepping-stone to the *Intellige ut credas* of Abelard. Wherefore let us first believe many things, if we would indeed finally know them.

A remarkable thinker, Henry Thomas Buckle, has committed himself, in the most unreserved manner, to opinions totally opposed to those which have here been brought forward. I refer to this fact, because objections, if well and forcibly urged, cannot fail, either by the darkness or the light that is in them, to form an instructive contrast, either to the light or the darkness that is in the opinions to which they are opposed. In the

* *Philosophical Essays*, pp. 6, 7.

first volume of his work, in a chapter entitled the Examination of the Method employed by Metaphysicians for Discovering Mental Laws, Buckle gives expression to views of which the following is a summary. Defining metaphysics "as that vast body of literature which is constructed on the supposition that the laws of the human mind can be generalized *solely* from the facts of individual consciousness," he undertakes to expose the erroneousness of the metaphysical scheme. Psychologists, he affirms, insist on the sufficiency of their method to make known the laws of mind. Since, however, this method consists in each observer studying the operations of his own intelligence, it must be quite unequal to the task of ascertaining the laws of all minds. "This is the direct opposite of the historical method; the metaphysician studying one mind, the historian studying many minds." The reply to this objection is contained in the preceding examination. Therein I profess, by the study of my own consciousness or intelligence, aided by the discoveries of others who have followed a similar method, to find out how necessary and universal truths are obtained, which is by a law of mind, itself a leading example of a necessary and universal truth. Now, by the historical method, which Buckle thinks should supersede the psychological method, one can, at the very most, only obtain general truths, truths which, let them be ever so general, never can attain to universality, infinite extension. In consequence of this, the historical is so far inferior to the psychological method, as a mere general truth is inferior in dignity and completeness to a universal truth. Indeed, in the reflective branch of mental science, this boasted historical method serves merely to illustrate, by numerous examples, a law of all minds, discovered by the psychological method.

Another forcible objection urged by this philosopher against the metaphysical scheme of investigating mental laws, is the notorious fact, that there are two methods of proceeding among philosophers, each of which leads to conflicting results. "According to the first method, the inquirer begins by examining his sensations. According to the other method, he begins by examining his ideas. These two methods always have led, and always must lead, to conclusions diametrically opposed to each other." "The idealist is compelled to assert, that necessary truths and contingent truths have a different origin. The sensationalist is bound to affirm that they have the same origin. The further these two schools advance, the more marked does their divergence become. They are at open war in every department of morals, of philosophy, and of art." This is certainly putting the question in a most objectionable light, but with what object? In order to expose the faultiness of the psychological method. "Under these circumstances, it is impossible to avoid a suspicion that there is some fundamental error in the manner in which these inquiries have been prosecuted." I fail, however, to see that it is not quite possible to avoid so illogical a course as to suspect that what has not yet been, cannot possibly be. The divergence, unfortunately, but, at the same time, inevitably existing among psychologists, is mainly due to the peculiar abstruseness of the subject which they seek to investigate. And if it be true, as Buckle says, that metaphysical systems "are diverging from each other with a velocity which seems to be accelerated by the progress of knowledge," may not the suspicion naturally arise that in this we simply have an instance of the fact, that the darkest hour is that before the dawn. It seems to me, however, that the darkest hour is passed, and that there never was a greater amount of convergence, merely beginning to disclose itself though it may be, among psychologists than at the present time. Fierce then as Buckle's assault is, there need not be the least misgiving as to its successful repulse. In confirmation of this statement, reference must be made to the indications contained in the preceding examination that it is quite possible to mediate between the rival systems of psychology, and that ere long it must be commonly acknowledged that every philosopher must adopt, not the one of these systems to the exclusion of the other, but both alike, because they comprise in their union the complex and sole method by which all science must be established. But Buckle goes so far as to say that it is impossible to bring about such an alliance. "Every system of metaphysics has been constructed according to one of these schemes; and this must always continue to be the case, because when the two schemes are added together, they include the totality of metaphysical phenomena. Each process is equally plausible; the supporters of each are equally confident; and by the very nature of the dispute, it is impossible that any middle term should be found; nor can there ever be an umpire, because no one can mediate between metaphysical controversies without being a metaphysician, and no one can be a metaphysician without being either a sensationalist or an idealist; in other words, without belonging to one of those very parties whose claims he professes to judge." This is certainly a clever argument, but the force of it rests on the assumption that because the two systems of psychology have always led to antagonistic results, they never can be made to harmonize. In proof, however, that these systems must always lead to opposite conclusions, Buckle has nothing to which to point but the past. They have always done so, and therefore must continue to do so. Now since I have already endeavoured to show the practicability of the alliance which Buckle here pronounces to be unattainable, I must, in this place, simply state that when there is no absolute impossibility in the case, it is wiser to suspend our judgment, than to pronounce a thing impossible. Let us not forget that we live in an age when many so called impossibilities have become common things.

---

